# Ligand binding properties of two *Brugia malayi* fatty acid and retinol (FAR) binding proteins and their vaccine efficacies against challenge infection in gerbils

**DOI:** 10.1371/journal.pntd.0006772

**Published:** 2018-10-08

**Authors:** Bin Zhan, Sridhar Arumugam, Malcolm W. Kennedy, Nancy Tricoche, Lu-Yun Lian, Oluwatoyin A. Asojo, Sasisekhar Bennuru, Maria Elena Bottazzi, Peter J. Hotez, Sara Lustigman, Thomas R. Klei

**Affiliations:** 1 Texas Children’s Hospital Center for Vaccine Development, Departments of Pediatric Tropical Medicine and Molecular Virology and Microbiology, National School of Tropical Medicine, Baylor College of Medicine, Houston, TX, United States of America; 2 Department of Pathobiological Sciences, LSU School of Veterinary Medicine, Louisiana State University, Baton Rouge, LA, United States of America; 3 Institute of Biodiversity Animal Health and Comparative Medicine, Graham Kerr Building, University of Glasgow, Glasgow, Scotland, UK; 4 Laboratory of Molecular Parasitology, Lindsley F. Kimball Research Institute, New York Blood Center, New York, NY, United States of America; 5 NMR Centre for Structural Biology, University of Liverpool, Crown Street, Liverpool, United Kingdom; 6 Laboratory of Parasitic Diseases, National Institute of Allergy and Infectious Diseases, Bethesda, MD 20892, United States of America; Task Force for Child Survival and Developmentorce for Global Health, UNITED STATES

## Abstract

Parasitic nematodes produce an unusual class of fatty acid and retinol (FAR)-binding proteins that may scavenge host fatty acids and retinoids. Two FARs from *Brugia malayi* (*Bm-*FAR-1 and *Bm-*FAR-2) were expressed as recombinant proteins, and their ligand binding, structural characteristics, and immunogenicities examined. Circular dichroism showed that r*Bm*-FAR-1 and r*Bm*-FAR-2 are similarly rich in α-helix structure. Unexpectedly, however, their lipid binding activities were found to be readily differentiated. Both FARs bound retinol and *cis-*parinaric acid similarly, but, while r*Bm*-FAR-1 induced a dramatic increase in fluorescence emission and blue shift in peak emission by the fluorophore-tagged fatty acid (dansyl-undecanoic acid), r*Bm*-FAR-2 did not. Recombinant forms of the related proteins from *Onchocerca volvulus*, r*Ov*-FAR-1 and r*Ov*-FAR-2, were found to be similarly distinguishable. This is the first FAR-2 protein from parasitic nematodes that is being characterized. The relative protein abundance of *Bm*-FAR-1 was higher than *Bm-*FAR-2 in the lysates of different developmental stages of *B*. *malayi*. Both FAR proteins were targets of strong IgG1, IgG3 and IgE antibody in infected individuals and individuals who were classified as endemic normal or putatively immune. In a *B*. *malayi* infection model in gerbils, immunization with r*Bm-*FAR-1 and r*Bm*-FAR-2 formulated in a water-in-oil-emulsion (®Montanide-720) or alum elicited high titers of antigen-specific IgG, but only gerbils immunized with r*Bm*-FAR-1 formulated with the former produced a statistically significant reduction in adult worms (68%) following challenge with *B*. *malayi* infective larvae. These results suggest that FAR proteins may play important roles in the survival of filarial nematodes in the host, and represent potential candidates for vaccine development against lymphatic filariasis and related filarial infections.

## Author summary

Human lymphatic filariasis (LF) and river blindness (onchocerciasis) are highly debilitating neglected tropical diseases. As with all parasitic nematodes, *Wuchereria bancrofti*, *Brugia malayi* and *Brugia timori*, the etiological agents of LF and *Onchocerca volvulus* the causative agent of river blindness, possess limited lipid metabolic pathways and hence rely on lipids scavenged from their human hosts. Two unusual lipid-trafficking proteins from *Brugia malayi* (*Bm-*FAR-1 and *Bm-*FAR-2) were expressed as recombinant proteins, and their ligand binding activities along with their structural characteristics were examined. Their immunogenicity in infected children and those who are classified as endemic normal or putatively naturally immune were also evaluated. Their immunogenicity and immunoprotective efficacies were also evaluated in a *B*. *malayi* gerbil infection model. The possible role these proteins play in the survival of filarial nematodes in the host, and their prospects of being candidates for vaccine against these highly pathogenic infections are discussed.

## Introduction

Human lymphatic filariasis (LF) and river blindness (onchocerciasis) are highly debilitating diseases in tropical developing countries with an estimated disease prevalence of 29.38 and 14.65 million cases that cause 1.2 and 0.96 million years lived with disability (YLD), respectively [1]. As with all parasitic nematodes, the etiological agents of LF such as *Wuchereria bancrofti*, *Brugia malayi* and *Brugia timori*, and that of river blindness, *Onchocerca volvulus*, possess limited lipid metabolic pathways and hence rely on lipids scavenged from their hosts [2]. Several structurally novel families of lipid-binding proteins in nematodes have been reported [3], including the fatty acid- and retinoid-binding protein family (FAR) that have been identified from many species of filarial nematodes including those from the genera *Onchocerca*, *Brugia*, *Wuchereria*, *Loa*, *Acanthocheilonema* and *Litomosoides* [4]. FAR proteins represent a structurally novel class of approximately 20 kDa lipid-binding proteins that are only found in nematodes [5], isoforms of which are known to be differentially expressed during development of parasitic and free-living species [5–7].

*Ov*-FAR-1 (previously known as Ov20 or *Ov*-RBD-1), a FAR protein from the filarial nematode *O*. *volvulus* was initially identified as a 20 kDa, structurally novel small helix-rich fatty acid and retinol (vitamin A)-binding protein secreted by the adult worm [8]. Soon thereafter, *Bm-*FAR-1, was described in *B*. *malayi*, and the two proteins were found to have similar secondary structure and ligand-binding characteristics [2, 4]. PCR-based strategies have since been used to isolate cDNAs encoding FARs from the filarial nematodes *Onchocerca*, *Brugia*, *Wuchereria*, *Loa*, *Acanthocheilonema* and *Litomosoides* [2, 4].

The ligand-binding properties of the filarial FAR proteins have been suggested to contribute, not only to their survival in the host, but also to pathogenesis in mammalian hosts [5, 8, 9]. These parasites appear to require retinoids and fatty acids for a variety of metabolic and developmental needs, including growth, development, differentiation, embryogenesis, and glycoprotein synthesis [2, 5, 10, 11]. FAR proteins have been shown to be released from the parasites into their hosts [2, 8, 12], suggesting that their FARs may also play an important role in modifying the local inflammatory and immunological environment of the surrounding host tissue by sequestering and/or delivering pharmacologically active lipids [5, 12]. Relevant to this hypothesis is the finding of high concentrations of retinol within onchocercal nodules [13]. Given the role of retinoids in vision, tissue differentiation and collagen synthesis [9], such sequestration of retinol might exacerbate vitamin A deficiency in infected humans, thereby contributing to the clinical manifestation of river blindness. It has been found that patients with onchocerciasis have lower serological level of vitamin A [14, 15].

The probable dependence of the filarial parasites on the FAR proteins for metabolic needs, and their potential roles in development and immune modulation of the host makes them pertinent targets for anthelmintic drugs and vaccine development. We therefore produced two FAR proteins from *B*. *malayi* in recombinant forms, biophysically characterised their hydrophobic ligand binding properties, and tested their immunogenicity and immunoprotective efficacy against infection with *B*. *malayi* infective larvae in gerbils. We found that despite their amino acid sequence relatedness and similar structural characteristics, and a precedent in another species, r*Bm-*FAR-1 and r*Bm-*FAR-2 could be clearly discriminated by their ligand-binding properties. We provide evidence that this disparity applies widely in filarial parasites. Immunization with recombinant *Bm-*FAR-1 conferred significant protection against infection in gerbils, but only when formulated in a water-in-oil adjuvant rather than alum. Our results indicate that *Bm-*FAR-1 is a candidate for a vaccine development against lymphatic filariasis, and related filarial helminthiases, and that *Bm*-FAR-2 might have to be further optimized before confirmed to being a promising vaccine candidate.

## Methods and materials

### Ethics statement

All the animals in this study were handled according to the National Institutes of Health (USA) guidelines and the animal experimentation was performed with prior approval from the Louisiana State University Institutional Animal Care and Use Committee under the protocol number 12–037.

The protocols used in all the population studies were approved by the Institutional Review Board (IRB) of the National Institute of Allergy and Infectious Diseases (Cook Islands studies, clinical protocol number 92-I-0155), the New York Blood Center's IRB (clinical protocol number 321 and 603–09), and by the National Institutes of Health (USA) accredited Institutional Review Board of the Medical Research Council Kumba, Cameroon (Kumba studies, clinical protocol number 001). Informed written consent was obtained from all adult subjects, and for children consent was obtained through both verbal assent and written consent from each subject’s legal guardian.

### Sequence analyses of *Bm-*FAR proteins

The encoded amino acid sequences of *Bm-*FAR-1 (GenBank accession# XP_001899742) and *Bm-*FAR-2 (XP_001900470) were aligned with FAR proteins from other nematodes including *Wuchereria bancrofti* (*Wb*-FAR-1:Q8WT54.2; *Wb*-FAR-2:EJW79208.1), *Onchocerca volvulus* (*Ov*-FAR-1: Q25619.1; *Ov*-FAR-2: ACT55269.1), *Loa loa* (*L1*-FAR-1: AAK84218.1; *Ll*-FAR-2: XP_003137038.2), *Dirofilaria immitis* (*Di*-FAR-1: nDi.2.2.2.t00119*; *Di*-FAR-2: nDi.2.2.2.t02086*); *Brugia pahangi* (*Bp*-FAR-1: Q8WT55.2; *Bp*-FAR-2: BPAG_0000817001-mRNA-1*), *Litomosoides sigmodontis* (*Ls*-FAR-1: Q8WT56.2; *Ls*-FAR-2: nLs.2.1.2.t01040-RA*), *Acanthocheilonema viteae* (*Av*-FAR-1: Q8MZJ8.1; *Av*-FAR-2: nAv.1.0.1.t08895-RA*), *Ascaris suum* (*As*-FAR-1: ERG87764.1; *As*-FAR-2: ERG85173.1), *Ancylostoma caninum* (*Ac*-FAR-1: AAM93667.1; *Ac*-FAR-2: ANCCAN_08556*), *Necator americanus (Na*-FAR-1 (PDB: 4UET_A, XP_013293708.1; *Na*-FAR-2: NECAME_14206*), *Toxocara canis* (*Tc*-FAR-1: KHN72925.1; *Tc*-FAR-2: KHN88420.1), *Haemonchus contortus* (*Hc*-FAR-: CDJ83169.1; *Hc*-FAR-2: HCOI00378700.t1*), *Caenorhabditis elegans* (*Ce*-FAR-1: NP_001254978.1; *Ce*-FAR-7 (PDB: 2W9Y_A), *Strongyloides ratti* (*St*-FAR-1: CEF68237.1), *Ostertagia ostertagi* (*Oo*-FAR-1: CAD20464.1). Similar sequences marked with [*] were obtained by BLAST searching nematode genomes databases at Wormbase (http://parasite.wormbase.org/index.html). A phylogenetic tree was generated using MEGALIGN from Lasergene 14, DNASTAR), and rendered using FigTree 1.4.3. For structural comparison of *Bm-*FAR-1 and *Bm-*FAR-2 with structure-defined orthologues *Na*-FAR-1 (PDB: 4UET_A) [16] and *Ce*-FAR-7 (PDB: 2W9Y_A) [17], the sequences were aligned with Clustal Omega and the secondary structural features were predicted based on the coordinates of *Na*-FAR-1 and *Ce*-FAR-7 using ESPript [18]. The structural theoretical ‘homology’ models of *Bm-*FAR-1 and *Bm-*FAR-2 were generated using Swiss model [19, 20].

### Production of recombinant *Bm-*FAR and *Ov*-FAR proteins

For production of recombinant proteins, the DNAs encoding *Bm-*FAR1 and *Bm-*FAR-2 (minus their signal peptides) were codon optimized for yeast preference and synthesized by GenScript (Piscataway, NJ, USA), and then inserted in-frame into the PichiaPink expression vector pPinkα-HC (Invitrogen, Carlsbad, USA) using XhoI/KpnI sites. The recombinant proteins with 6His-tag at C-terminus were induced in PichiaPink strain 4 (with protease PEP4 and PRB1 deleted to reduce degradation) with 5% methanol and purified with immobilized metal affinity chromatography (IMAC) as previously described [21, 22]. Due to the high levels of glycosylation of the yeast expressed *Bm-*FAR-2 recombinant protein, it was re-cloned in *E*. *coli* expression vector pET41a (Novagen, USA) with glutathione-S-transferase-tag deleted (NdeI/XhoI), and then transformed into BL21(DE3) cells (Novagen, USA). Recombinant *Bm-*FAR-2 protein (r*Bm-*FAR-2) was induced with 1 mM IPTG and purified with IMAC as described [23]. The r*Bm*-FAR-1 expressed in yeast, and the r*Bm*-FAR-2 expressed in *E*. *coli* were used for subsequent binding activity and vaccination experiments. The recombinant proteins of FAR orthologues in *O*. *volvulus* (r*Ov*-FAR-1 and r*Ov*-FAR-2) were produced in *E*. *coli* using similar procedures as r*Bm*-FAR-2.

### Parasite stage-specific expression of *Bm*-FAR proteins

Stage-specific proteomic expression patterns for the adult *B*. *malayi* male (AM) and female (AF) parasites, microfilariae (MF), immature MF (intrauterine stages; UTMF), and the third-stage larvae (L3) were derived from the *B*. *malayi* somatic proteomes [24], and normalized using normalized spectral abundance factor (NSAF), where the relative abundance of a protein in a sample was calculated by: $NSAF=\frac{{\left(\frac{Spectra}{Length}\right)}_{p}}{\sum_{p=1}^{n}{\left(\frac{Spectra}{length}\right)}_{p}}$.

The *in-vitro* derived L4 [25] were lysed in lysis buffer, dialyzed, desalted, and digested with trypsin. The cation-exchange liquid chromatography fractionation of tryptic peptides was analyzed by nanobore reverse-phase liquid chromatography (RPLC-MS/MS). Proteins were identified by searching the spectral data using PEAKS 7 using a combined database of *B*. *malayi* (Wormbase WBPS9 ver) and its endosymbiont *Wolbachia* (*wBm*).

### Circular dichroism (CD)

A Jasco-J1100 CD Spectrophotometer was used for measurements in the far ultraviolet region (UV), from 190 to 260 nm. Spectra were recorded at protein concentrations of approximately 0.1–0.2 mg ml^-1^ in a cuvette of 0.2 mm path length in a temperature-controlled cell holder at 25°C. Spectra were monitored with a 0.2-nm step with 10 averages per step. Samples were prepared in 20 mM Tris, 20 mM NaCl, pH 6.8 buffer, and all spectra were baseline corrected by subtraction of the spectra for buffer alone. Analysis of the CD spectra was undertaken using the CDSSTR within the Dichroweb program [26, 27].

### Fluorescence-based lipid binding assays

Recombinant FAR proteins of *B*. *malayi* and *O*. *volvulus* (r*Bm-*FAR-1, r*Bm-*FAR-2, r*Ov*-FAR-1 and r*Ov*-FAR-2), were used in fluorescence-based lipid binding assays as previously described [5, 12, 28]. The concentrations of the proteins were estimated by absorbance at 280 nm (correcting for any untoward absorbances at 230 and 260 nm), using theoretical extinction coefficients based on their amino acid compositions, and calculated using the ProtParam tool online at http://web.expasy.org/protparam. The protein concentrations of all were calculated to be at approximately 4 mg ml^-1^. Lipid binding was detected spectrofluorometrically, using all-*trans* retinol, or the fluorescent fatty acid analogue 11-((5-dimethylaminonaphthalene-1-sulfonyl)amino)undecanoic (DAUDA), which bears the environment-sensitive dansyl fluorophore, or with the intrinsically fluorescent *cis*-parinaric acid (cPnA), all as described before [5]. DAUDA and cPnA were obtained from Molecular Probes/Invitrogen (Renfrew, UK). All-*trans*-retinol and oleic acid were obtained from Sigma (Poole, Dorset, UK). The excitation wavelengths were 345 nm, 350 nm, and 319 nm for DAUDA, retinol, and cPnA, respectively, all of which were at final concentrations of approximately 1 μM, 4 μM, and 4 μM, respectively, in 2 ml phosphate buffered saline (PBS) pH 7.2 in a quartz fluorescence cuvette. Binding of a non-fluorescent ligand was detected by a reversal of fluorescence emission enhancement elicited by a test protein upon addition of the ligand to a preformed DAUDA:protein complex. Note that r*Bm-*FAR-2 is larger than r*Bm-*FAR-1, so at equivalent w/v concentrations their molarities will differ. Also, we cannot assume that the proportion of properly folded and active protein in each protein sample is equivalent, or that there is no interference from resident ligand(s) derived from the bacteria in which the proteins were produced despite routine detergent and lipid depletion–our experience is that complete removal of resident lipids from nematode lipid-binding proteins requires methods such as reverse-phase chromatography [29], which was not carried out in the present study. The fluorescence spectra are uncorrected and were analyzed using MICROCAL ORIGIN software.

### Antibody responses to *Bm*-FARs and *Ov*-FARs in infected, endemic normal and/or putatively immune humans

The serum samples used for the present studies were from a repository from two distinct clinical studies in which plasma samples were collected from two different populations. First, two population studies performed in 1975 and 1992 in the island of Mauke in the Southern Cook Islands, a region endemic for the filarial parasite *W*. *bancrofti*. The characteristics of the population studied have been described in detail in previous publications [30, 31]. Second, a clinical study performed between 1995 and 2000 in the Kumba region of southwest Cameroon, an area of hyperendemicity for onchocerciasis. The characteristics of this population have also been described previously [32, 33]. Antibody responses to r*Bm*-FAR-1 and r*Bm*-FAR-2 were tested in children (age 2–9) that were infected (INF; n = 21; microfilaremic and/or positive for circulating filarial antigen) or classified as uninfected or endemic normal (EN; n = 22). Antibody responses to r*Ov*-FAR-1 and r*Ov*-FAR-2 were tested in putatively immune (PI; n = 12; age 5–59), and age and sex match infected (INF; n = 30; 6–41) individuals.

Sera obtained from the individuals were analyzed for IgG1, IgG3 and IgE isotype antibody responses using recombinant *Bm*-FARs and *Ov*-FARs using ELISA protocols established for testing antibody responses to filarial recombinant proteins [30–32, 34–36], with some modification. Briefly, each recombinant protein (1 μg/ml) was used to coat the wells of ELISA plates, and sera at a 1:100 (IgG1 and IgG3) or at 1:50 (IgE) dilution were applied. The bound IgG1 or IgG3 antibodies were detected by using a 1:1,000 dilution of monoclonal antibodies against human IgG1 and IgG3 subclasses (Hybridoma Reagent Laboratory, Kingsville, MD). This step was followed by incubation with a 1:1,250 dilution of horseradish peroxidase (HRP)-conjugated rabbit anti-mouse immunoglobulins (Kierkegaard & Perry Laboratories, Inc., Gaithersburg, Md.). Tetramethylbenzidine (Sigma) was used as the chromogen, and the optical density (OD) was read at 450 nm. For IgE responses, 1:1,000 dilution of monoclonal biotin conjugated anti-human IgE antibody (Hybridoma Reagent Laboratory, Kingsville, MD) followed by incubation with 1:1,000 Streptavidin -HRP (Invitrogen) and 0.4 mg/ml of o-Phenylenediamine dihydrochloride (Sigma) dissolved in phosphate citrate buffer with 0.05%H_2_O_2_. The optical density (OD) was read at 492nm. A pool of 10 normal human sera from de-identified New York blood donors was used as a negative control, and for setting up the cutoff; mean OD ± 3X SD.

### Animals and parasites

Mongolian gerbils, obtained from Charles River (Wilmington, MA, USA) at 8–10 weeks of age, were maintained on standard rodent diet and water *ad libitum*. Infective third-stage larvae (L3) of *B*. *malayi* were recovered from infected *Aedes aegypti* mosquitoes using the previously described Baermann technique [37]. All the animals in experimental and control groups received 100 *B*. *malayi* L3 subcutaneously in 0.5 ml of RPMI-1640 medium.

### Immunization and challenge infection

The r*Bm-*FAR-1 and r*Bm-*FAR-2 were formulated with alum (Rehydragel LV, General Chemical, NJ) at ratio of 1:8 (w/w) (25 μg of recombinant protein was mixed with 200 μg of alum) for 30 min at room temperature with shaking [21]. The virtually complete binding of recombinant FAR proteins to alum was confirmed by SDS-PAGE. Ten 8–10 weeks old male Mongolian gerbils in each group were immunized intraperitoneally (IP) with 25 μg of r*Bm-*FAR-1 formulated with alum as described above or emulsified with Montanide-720 (Seppic, Paris, France) at ratio of 30/70 (v/v) in a total volume of 100 μl. Gerbils were boosted twice with the same dose at two-week intervals. Another two groups of gerbils were intraperitoneally injected with PBS formulated with the same adjuvants (alum or Montanide-720) as controls using the same regimen. Sera were collected one week after each immunization. IgG responses to r*Bm-*FAR-1 or to r*Bm-*FAR-2 were measured as previously described [38]. Serial dilutions of gerbil serum were made, and total antigen-specific IgG response to recombinant antigens were evaluated for each group.

Two weeks after the third immunization, all gerbils in each group were challenged subcutaneously with 100 infective L3 of *B*. *malayi*. Necropsy was performed 42 days post-infection and adult worms were recovered from different body regions of gerbils as described previously [38, 39]. Protective immunity induced by immunization is expressed as the percentage reduction in worm burden as calculated by subtracting the average number of worms recovered in immunized gerbils from the average number of worms recovered from the adjuvant control gerbils divided by average worms recovered from control gerbils and then multiplied by 100.

### Statistics

Analysis of human antibody response data was carried out using the non-parametric Mann-Whitney test to examine statistical significance between the immune responses of infected and EN (uninfected) and putatively immune individuals to FAR proteins. For the immunization experiments in gerbils, every experiment comprised 10 gerbils per group, and statistical analysis between antigen-vaccinated groups and adjuvant control groups were performed using the Mann-Whitney U test in GraphPad Prism 6 (GraphPad Software, San Diego, California USA). Data were expressed as means ± standard deviation. A value of *p* < 0.05 was considered as statistically significant.

## Results

### Sequence and structural analysis of *Bm-*FAR-1 and *Bm-*FAR-2

*Bm-*FAR-1 was the first fatty acid and retinol binding protein identified in *B*. *malayi* with α-helix-rich structure and lipid binding activity similar to *Ov*-FAR-1, its orthologue from *O*. *volvulus* [2, 5]. BLAST searching of the *B*. *malayi* genome database [40] revealed a paralogous FAR protein, here designated *Bm-*FAR-2. Both *Bm-*FAR-1 and *Bm-*FAR-2 contain the consensus casein kinase II phosphorylation site which has been found in the same position in the amino acid sequence in other FAR proteins [2, 7, 41]. The present study is the first to our knowledge that characterizes a FAR-2 protein from a parasitic nematode.

**Fig 1:**
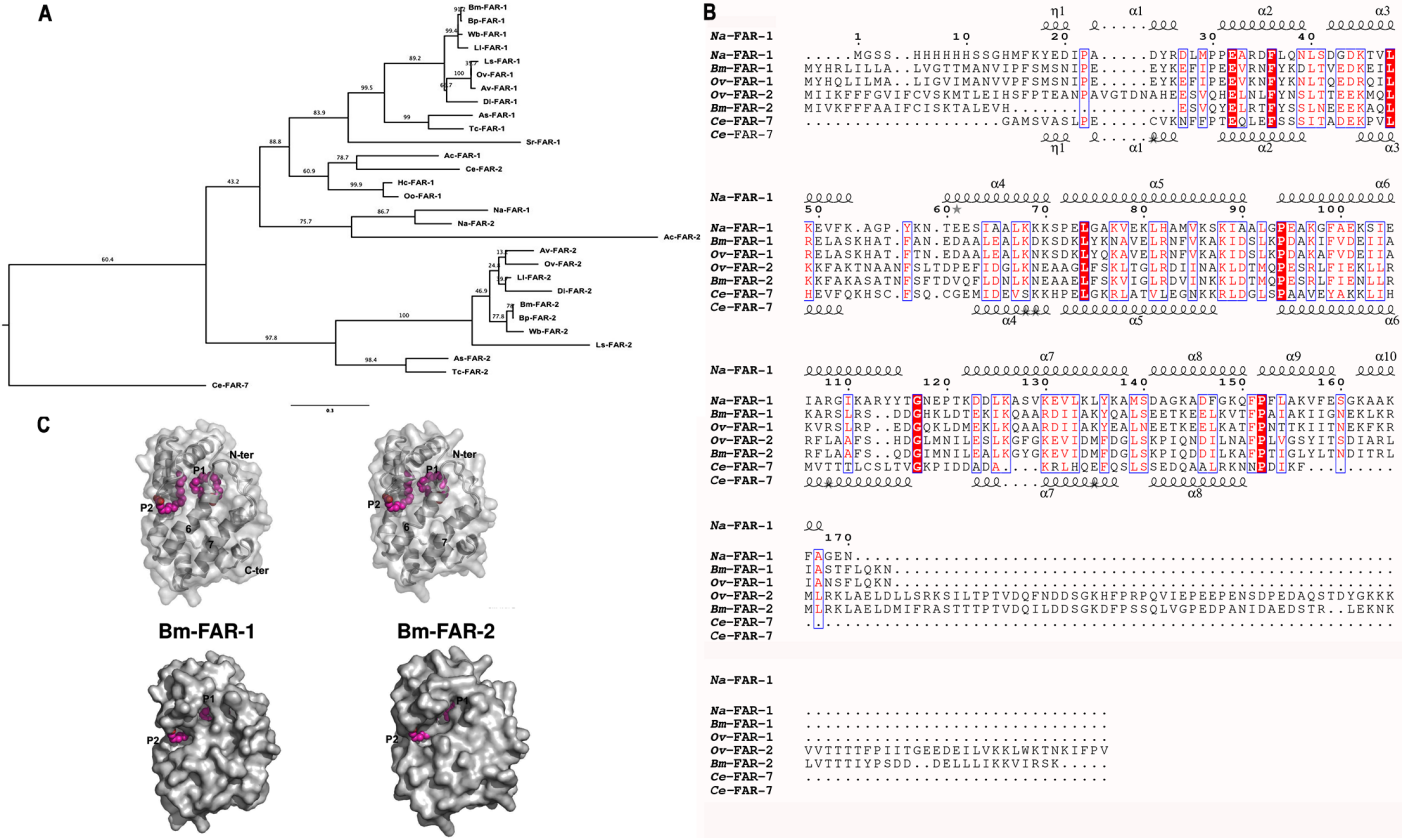
Sequence and structural analysis of *Bm-*FAR-1 and *Bm-*FAR-2. (**A)** Phylogenetic tree of *Bm*-FAR-1 and *Bm*-FAR-2 and their homologues in other nematodes, with bootstrap values (**B)** Structure based sequence comparison of *Bm-*FAR-1 and *Bm-*FAR-2, as well as *Ov*-FAR-1 and *Ov*-FAR-2, with *Na*-FAR-1 and *Ce*-FAR-7. The sequences are aligned with Clustal Omega and the secondary structural features are illustrated with the coordinates of *Na*-FAR-1 and *Ce*-FAR-7 using ESPript [52]. The different secondary structure elements shown are alpha helices (α), 3_10_-helices (η), beta strands (arrows), and beta turns (TT). Identical residues are shown in solid red, and conserved residues are in red. Black box indicates the conserved casein kinase II phosphorylation site. The N-glycosylation site at 53-NFS for *Bm*-FAR-2 and *Ov*-FAR-2 is underlined. The amino acid sequence identity of orthologues between *B*. *malayi* and *O*. *volvulus* is shown at the end of sequence alignment. (**C)** Predicted tertiary structures of *Bm*-FAR-1 and *Bm*-FAR-2 showing retinol-binding pocket (P1) and fatty acid binding pocket (P2). The palmitate molecules from the *Na*-FAR-1 structure are modeled into the pockets as magenta spheres. Top panel shows transparent surfaces of the models and the retinol-binding pocket P1 is formed by helices α2 and α6, which are well conserved. The fatty acid binding cavity P2 is formed by helices α4 and α5, which are poorly conserved. The bottom panel of the solid surface plot shows that the retinol-binding pockets (P1) are similarly located and oriented in both models, whereas the fatty acid binding pockets (P2) are smaller and less accessible in *Bm-*FAR-2.

Phylogenetic analysis shows that *Bm-*FAR-1 and *Bm-*FAR-2 fall into different clades of nematode FARs. *Bm-*FAR-1 is closely related to putative FAR-1 homologues from other filariae such as *B*. *pahangi* (*Bp*-FAR-1, 99% amino acid identity), *W*. *bancrofti* (*Wb*-FAR-1, 97%), and *O*. *volvulus* (*Ov*-FAR-1, 82%) [2]. *Bm-*FAR-2 shares only 27% amino acid sequence identity to *Bm-*FAR-1 and possesses an unique N-linked glycosylation site at 53-NFS and 70 additional C-terminus amino acids, but instead is more similar to FAR-2 orthologues from closely related species (88% sequence identity with *Wb*-FAR-2, and 63% with *Ov*-FAR-2) (Fig 1A and 1B). Molecular structures of two nematode FARs have been reported, one from *Necator americanus* (*Na*-FAR-1 by protein nuclear magnetic resonance (NMR) and x-ray crystallography; PDB accessions 4UET and 4XCP, respectively) [16], and another from *Caenorhabditis elegans* (*Ce*-FAR-7, by x-ray crystallography; PDB: 2W9Y) [17]. Notably, the molecular structure of *Na*-FAR-1 revealed a surface-proximal and a deeper binding pocket for hydrophobic ligands that are accessible to palmitate molecules.

Both *Bm-*FAR-1 and *Bm-*FAR-2 share less than 32% amino acid sequence identity with either *Na*-FAR-1 or *Ce*-FAR-7. However, these structures proved to be useful for generating theoretical models of *Bm*-FAR-1 and *Bm*-FAR-2 using Swiss-Model [19, 20]. The structure-based comparison of the sequences using ESPript [18] reveals that the amino acids in the binding pockets of *Bm-*FAR-1 are more similar to *Na*-FAR-1, while those of *Bm-*FAR-2 are more similar to *Ce*-FAR-7 (Fig 1B). The two distinct pockets in filarial nematode FARs are evident in the predicted structures (Fig 1B & 1C). The deeper pocket (P1) may bind retinol whereas the exterior pocket (P2) may bind fatty acids [16, 17]. The putative retinol-binding pocket P1 is formed by helices α2 and α6, which are well conserved (Fig 1B). The putative fatty acid binding cavity P2 is formed by helices α4 and α5, which are poorly conserved (Fig 1B). While the putative retinol-binding pocket (P1) is similarly located and oriented in both models, the potential fatty acid binding cavity (P2) is smaller and less accessible in *Bm-*FAR-2 than *Bm-*FAR-1 (Fig 1C), which may explain the differences between the two proteins in their binding of one of our fluorescent fatty acid probes (see below).

### Higher abundance of *Bm*-FAR-1 in different stages of *B*. *malayi*

**Fig 2:**
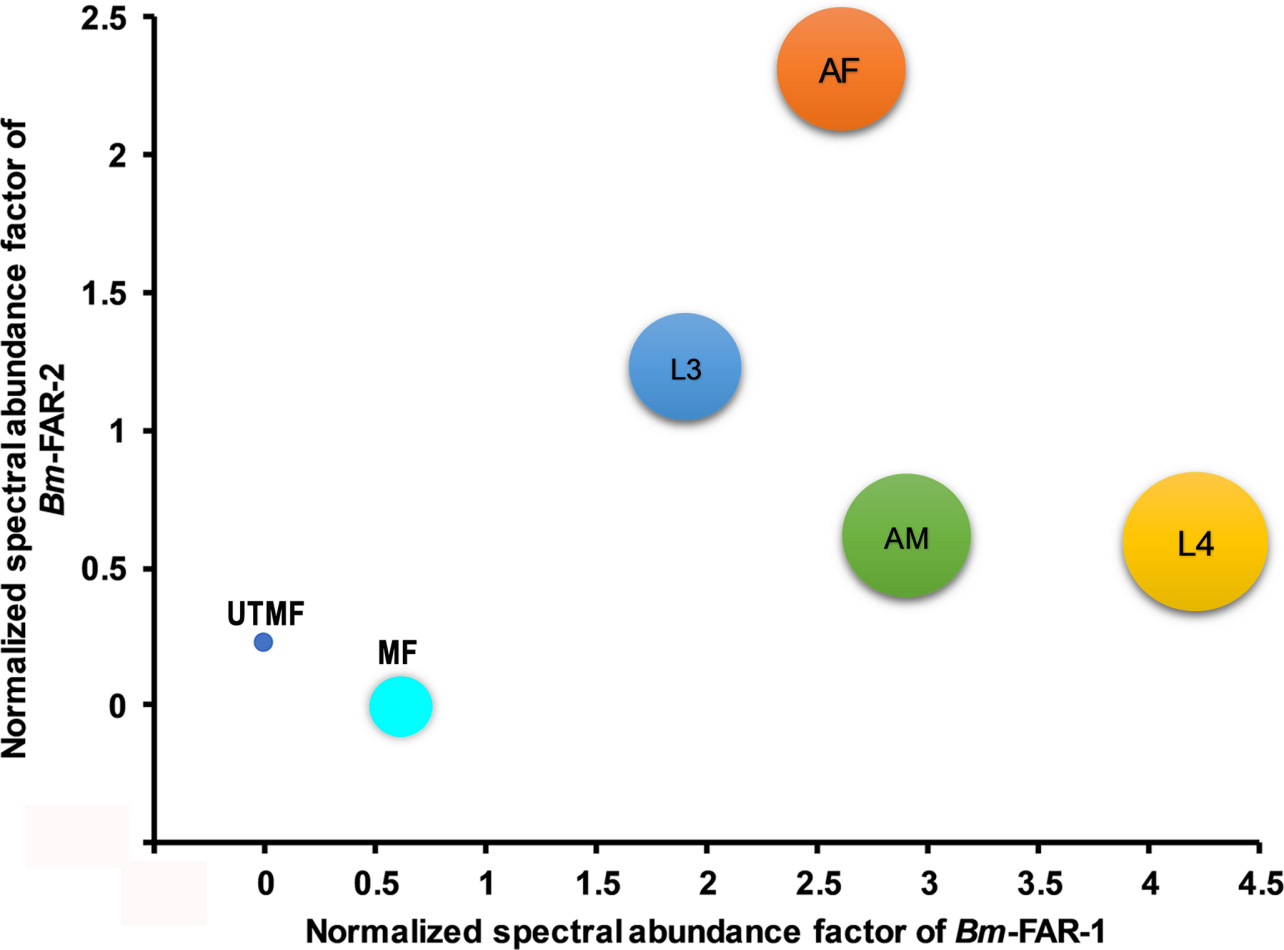
Bubble plot showing the normalized spectral abundance levels of BmFAR-1 and BmFAR-2 across the mature microfilaria (MF), immature microfilariae (UTMF), L3 larvae, *in-vitro* derived L4 larvae (L4), adult female (AF) and male (AM) worms based on proteomic analysis. The size of the bubble is proportional to the abundance of Bm-FAR-1.

The normalized spectral abundance levels of *Bm*-FAR-1 and *Bm*-FAR-2 in the lysates of different stages of *B*. *malayi* showed that the mature microfilariae (MF) and the immature uterine microfilariae (UTMF) only exhibited very low-levels of either *Bm*-FAR-1 or *Bm*-FAR-2. Overall, *Bm*-FAR-1 was more abundant than *Bm*-FAR-2 across all the stages, with the adult male worm and L4 stages exhibiting the highest abundance (4-fold and ~7-fold respectively) (Fig 2). The similar abundances of both proteins in the adult female (containing both mature and immature MFs) suggest that the source of the proteins is primarily the adult female.

### Production of recombinant *Bm-*FAR-1 and *Bm-*FAR-2 proteins in *E*. *coli*

**Fig 3:**
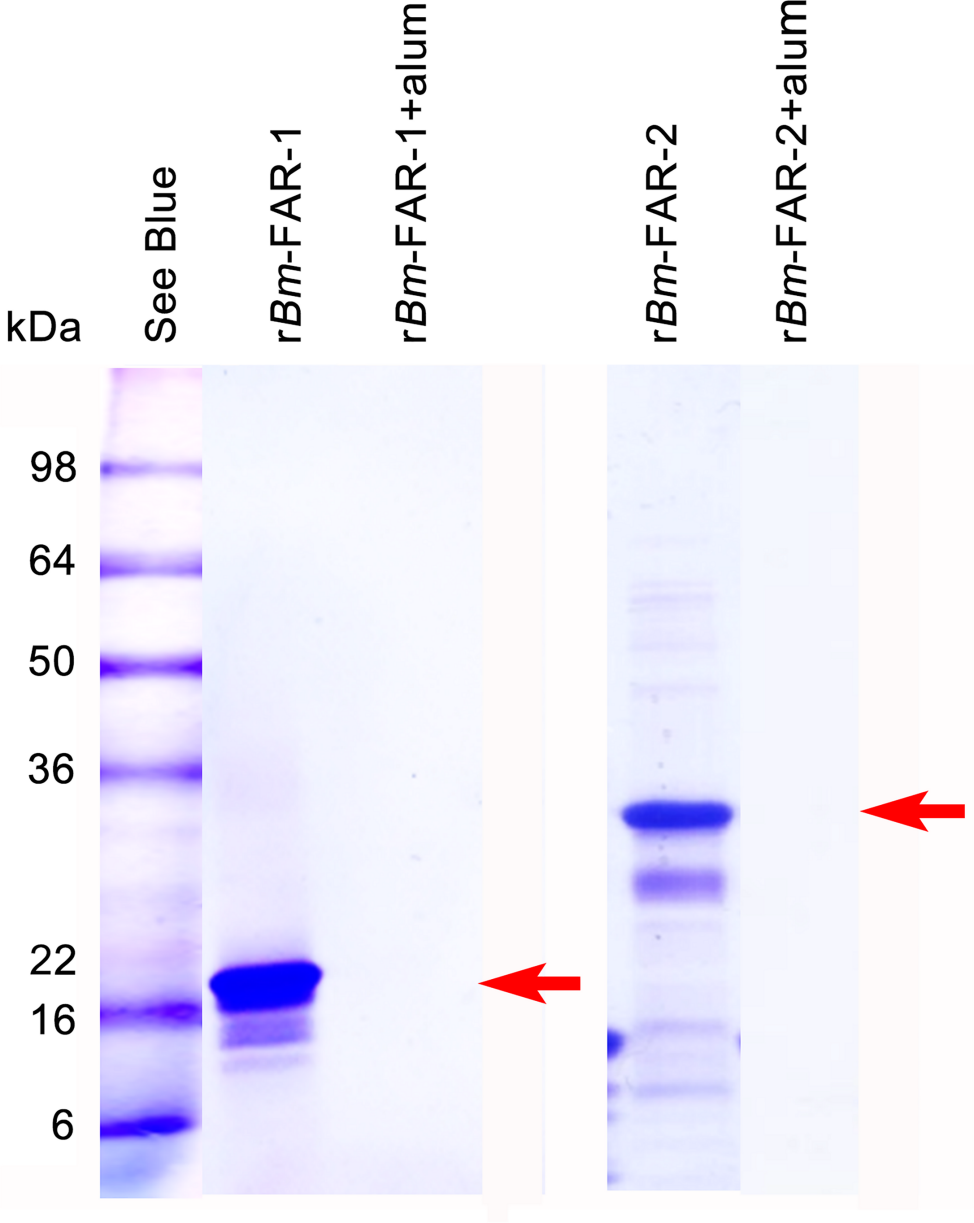
SDS-PAGE of yeast expressed r*Bm-*FAR-1 and *E*. *coli* expressed r*Bm-*FAR-2 and their binding to alum (Rehydrogel). Two μg of recombinant proteins were loaded in each lane (red arrow). The same amount of recombinant proteins was absorbed with Rehydrogel and the absorbed supernatant was loaded to adjacent well.

The r*Bm*-FAR-1 protein was expressed in yeast and r*Bm*-FAR-2 was expressed in *E*. *coli*. The purified r*Bm-*FAR-1 and r*Bm*-FAR-2 were observed to migrate at their corresponding molecular sizes of ~19 kDa and 27kDa, respectively, on SDS-PAGE (Fig 3). The additional lower molecular size bands for r*Bm*-FAR-2 were reactive to anti-His antibody and so were likely degradation products of the protein (S1 Fig). Both r*Bm-*FAR-1 and r*Bm-*FAR-2 were essentially fully absorbed by the alum-based adjuvant (Fig 3).

### Secondary structure content of *Bm-*FAR-1 and *Bm-*FAR-2

**Fig 4:**
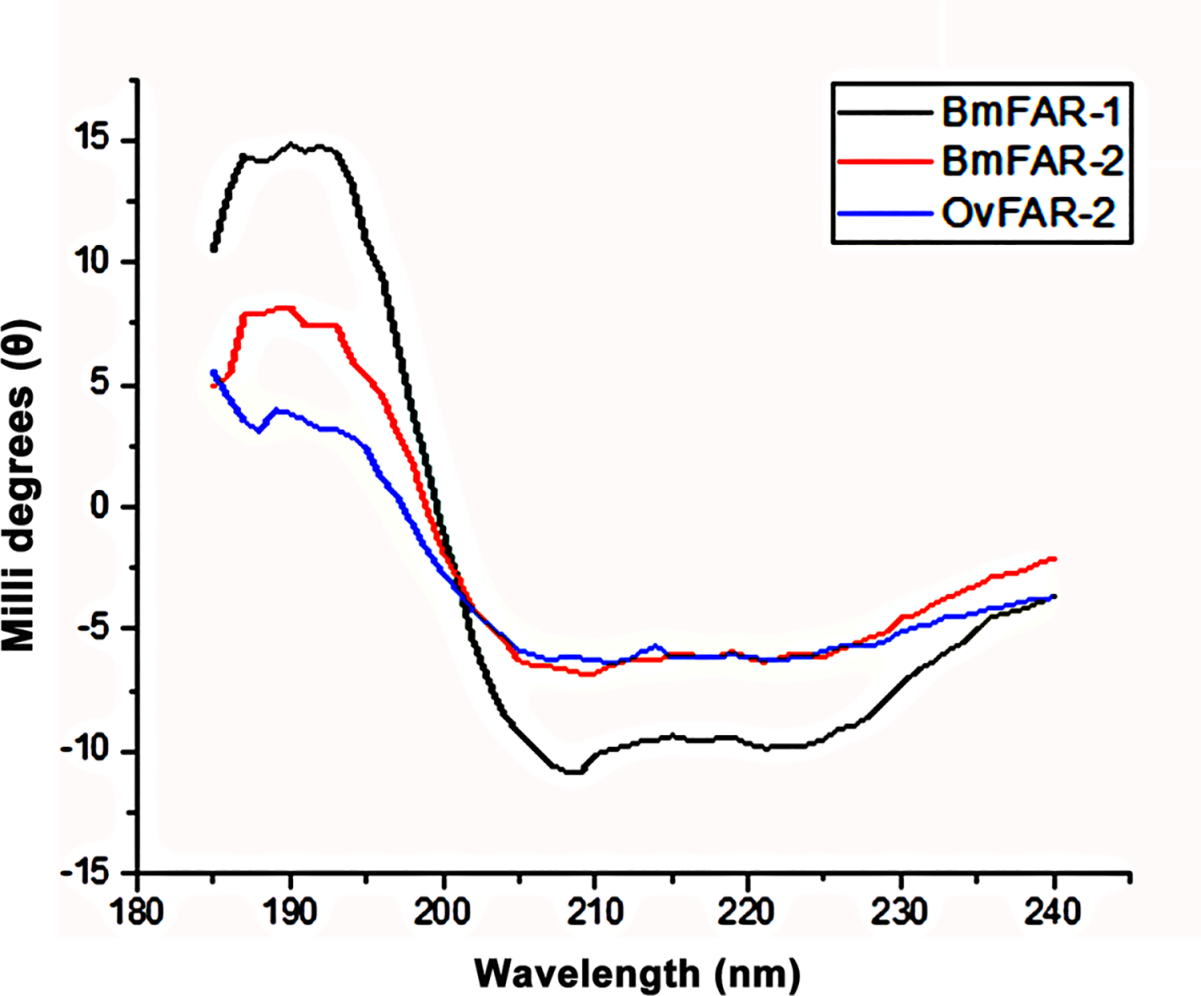
Circular dichroism spectra of r*Bm-*FAR-1, r*Bm-*FAR-2 and r*Ov*-FAR-2 in 20 mM Tris, 20 mM NaCl, pH 6.8 TBS, 25 ^o^C.

Both r*Bm-*FAR-1 and r*Bm-*FAR-2 were examined using circular dichroism, which showed that both proteins are α-helix-rich, with negative molar ellipticity (Ɵ) values at 222 nm and 208 nm, and a positive value at 193 nm [42]. The calculated helicity for both proteins is 75%, while the control protein r*Ov*-FAR-2, the presumed orthologue of *Bm-*FAR-2 from *O*. *volvulus*, contained 67% helicity (Fig 4). The high degree of helical structure for both *Bm-*FAR-1 and *Bm-*FAR-2, as estimated by CD, are similar to the predictions from homology modelling and sequence analyses, which indicate a predominance of α-helices (Fig 1B).

### Lipid binding by r*Bm-*FAR-1 and r*Bm-*FAR-2

**Fig 5:**
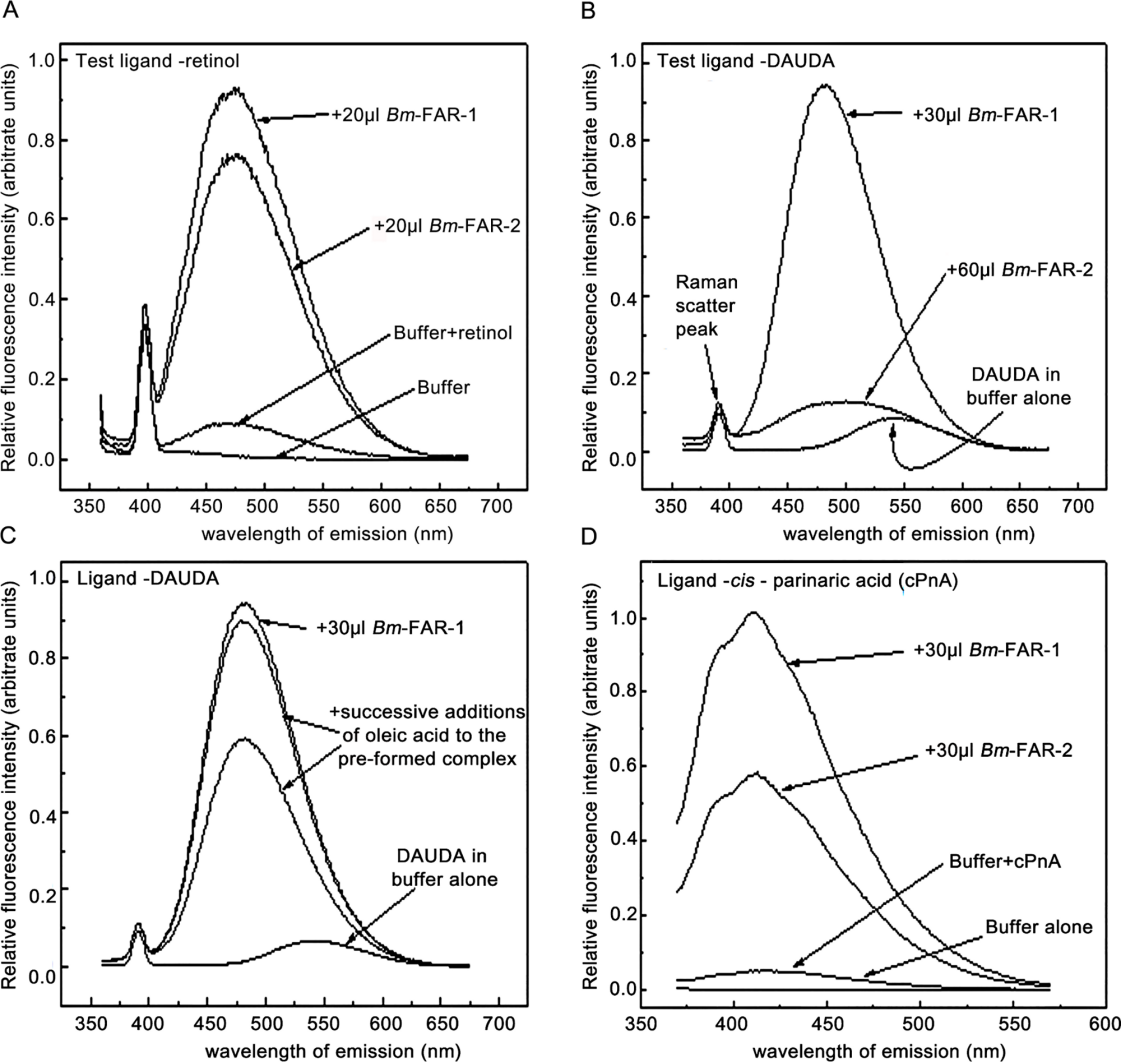
Lipid binding properties of r*Bm-*FAR-1 and r*Bm-*FAR-2. Fluorescence emission spectra were recorded, and spectrofluorimetry conditions set, as described in Materials and Methods. **(A)** Retinol in ethanol was added to 2 ml PBS, pH 7.2 (to create an approximately 4 μM solution of retinol) alone, or 2 ml PBS containing r*Bm-*FAR-1 or r*Bm-*FAR-2. Both proteins clearly bind retinol. (**B)** When *rBm-*FAR-1 and r*Bm-*FAR-2 were added to 2 ml of an approximately 1 μM solution of DAUDA in PBS, *Bm-*FAR-1 elicited a dramatic increase and blue shift in DAUDA fluorescence emission, whereas *Bm-*FAR-2 did so to a minimal extent. (**C)** Addition of oleic acid to pre-formed *Bm-*FAR-1:DAUDA complexes results in competitive displacement of DAUDA from the protein binding site, indicating that there exists a binding site with preference for natural fatty acids. (**D)** Both proteins bind *cis*-parinaric acid (cPnA), albeit yielding different amplitudes of change in cPnA emission, but this nevertheless indicates that both proteins bind fatty acids. Similar experiments were carried out with the orthologues of these proteins from the river blindness parasite *O*. *volvulus* (r*Ov*-FAR-1 and r*Ov*-FAR-2) and the results were directly comparable to results with the *Bm-*FARs (see S2 Fig).

The fatty acid and retinol binding properties of r*Bm-*FAR-1 and r*Bm-*FAR-2 were investigated using a fluorescence-based lipid binding assay. Addition of retinol to either r*Bm-*FAR-1 or r*Bm-*FAR-2 resulted in substantial increases in fluorescence emission, indicative of entry of retinol into an apolar protein binding site (Fig 5A). When the fluorescent fatty acid analogue, 11-(dansylamino) undecanoic acid (DAUDA), was used as test ligand, r*Bm-*FAR-1 produced a large increase in DAUDA emission and a substantial blue-shift in the wavelength of peak fluorescence emission from 542 to 483 nm (Fig 5B). This degree of blue shift in DAUDA’s emission is indicative of entry into a highly apolar environment and isolation from solvent water. In contrast, r*Bm-*FAR-2 produced a mild enhancement in DAUDA fluorescence, indicating that r*Bm-*FAR-2 interacts poorly with DAUDA, or that it does bind this ligand but in a way that leaves the attached dansyl fluorophore still in a polar environment (Fig 5B). Addition of oleic acid to preformed DAUDA: r*Bm-*FAR-1 complexes resulted in a significant decrease in the fluorescence intensity, indicating displacement of DAUDA from the binding site into polar solvent, and probable congruence between the binding sites for the two lipids (Fig 5C). Both r*Bm-*FAR-1 and r*Bm-*FAR-2 bound cPnA, which is an intrinsically fluorescent fatty acid that does not bear a bulky attached fluorophore (as is the case for DAUDA) (Fig 5D). The enhancement of cPnA’s fluorescence emission with r*Bm-*FAR-2 was less than that with r*Bm-*FAR-1, which may be due to fundamental differences in their hydrophobic ligand-binding properties, or that the former protein’s population may not have been proportionately as well folded as the latter’s (despite their retinol- and cPnA-binding activities being similar here), or that the environment of their binding sites for cPnA are slightly different. Essentially, however, both r*Bm-*FAR-1 and r*Bm-*FAR-2 bind fatty acids and retinol (and possibly other hydrophobic ligands), but can be discriminated by activity with DAUDA.

In parallel experiments, r*Ov*-FAR-1 and r*Ov*-FAR-2, which are presumed orthologues of r*Bm-*FAR-1 and r*Bm-*FAR-2, respectively, behaved in the fluorescence assays similarly to their counterpart *Bm-*FARs (S2 Fig).

### Antibody responses to r*Bm*-FARs and r*Ov*-FARs in infected, endemic normal and putatively immune humans

**Fig 6:**
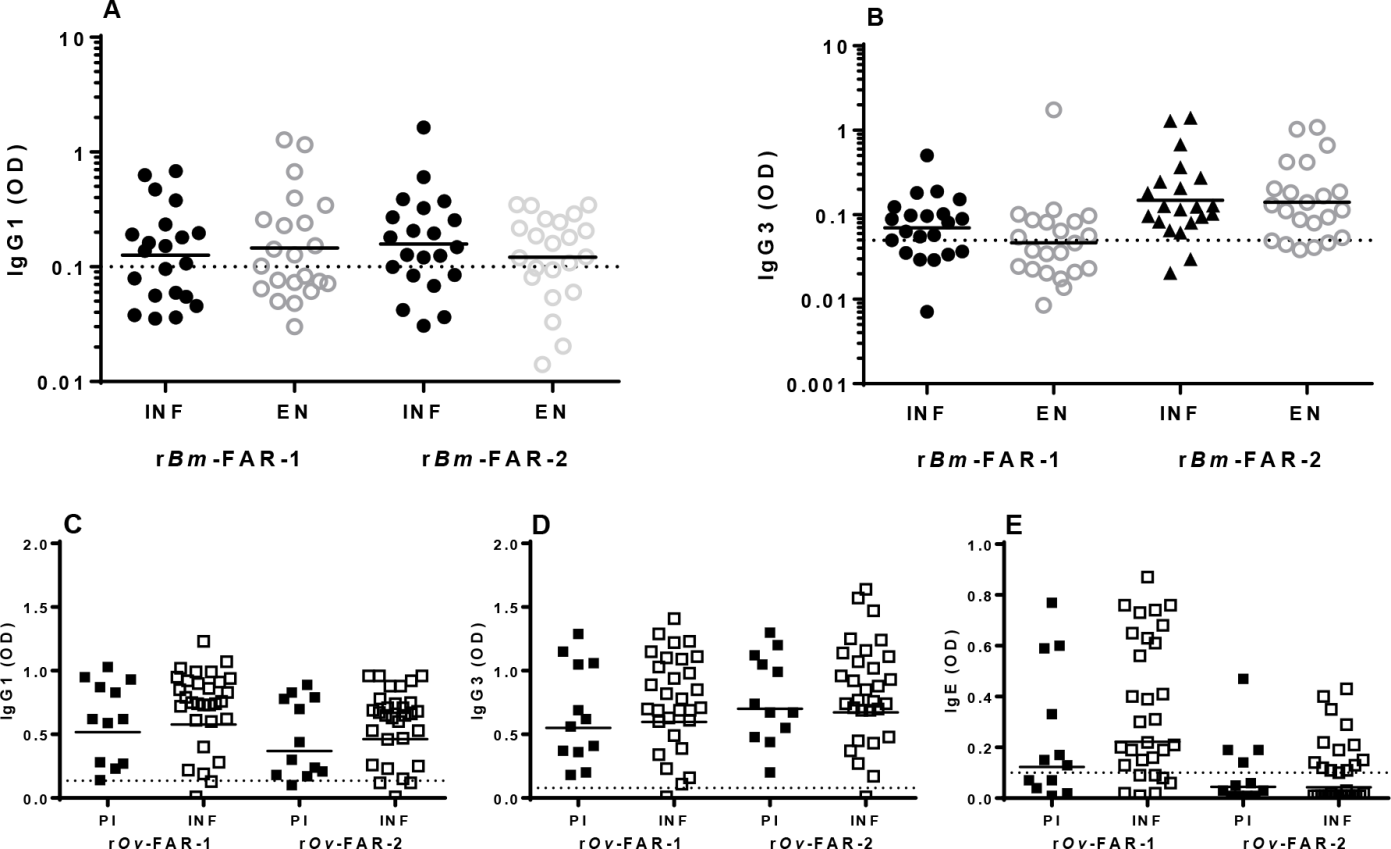
IgG1, IgG3 and IgE antibody responses against *Bm-*FARs and *Ov*-FARs in human populations living in filariae endemic regions. Anti-r*Bm*-FAR-1 and r*Bm*-FAR-2 IgG1 (**A**) and IgG3 (**B**) responses in children that were infected (INF) or uninfected (EN) who lived in Cook Islands, an endemic region for lymphatic filariasis. Anti-r*Ov*-FAR-1 and r*Ov*-FAR-2 IgG1 (**C**), IgG3 (**D**) and IgE (E) responses in putatively immune (PI) and infected (INF) individuals who lived in onchocerciasis highly endemic area in Cameroon. The ODs obtained with a pool of 10 control normal human sera samples at 1:100 (IgG1 and IgG3) or 1:50 (IgE) dilution were used to calculate the cutoff for each assay based on mean OD ± 3X SD (dotted line): 0.1 for anti-r*Bm*-FAR-1 and anti-r*Bm*-FAR-2 IgG1; 0.05 for anti-r*Bm*-FAR-1 anti-r*Bm*-FAR-2 IgG3; 0.135 for anti-r*Ov*-FAR-1 and anti-r*Ov*-FAR-2 IgG1; 0.08 for anti-r*Ov*-FAR-1 and anti-r*Ov*-FAR-2 IgG3; and 0.1 for anti-r*Ov*-FAR-1 and anti-r*Ov*-FAR-2 IgE. The cutoff was used to calculate the number of responders, individual having IgG1, IgG3 or IgE OD values more than mean + 3X SD of normal human sera. Analysis was done using the Mann-Whitney test.

When the r*Bm*-FARs antigen-specific IgG1 and IgG3 responses in sera from infected (INF) or exposed but uninfected/endemic normal (EN) children living in *W*. *bancrofti* endemic region, and the r*Ov*-FARs antigen-specific IgG1, IgG3 and IgE responses in sera from *O*. *volvulus* putatively immune (PI) or infected individuals (INF) were performed (Fig 6), it appears that both FAR proteins are immunogenic in all groups tested; no statistical differences were observed between the INF and EN children living in *W*. *bancrofti* endemic region (Fig 6A and 6B) or the PI and INF individuals living in onchocerciasis endemic region (Fig 6C and 6D).

However, it appears that both proteins are more immunogenic in the *O*. *volvulus* endemic population as the number of IgG1 and IgG3 responders to r*Ov-*FAR-1 and to r*Ov-*FAR-2 antigens was similarly high in both the PI and INF groups (>92% IgG1 responders to both antigens; >93% IgG3 responders to both antigens). The IgE responses were more modest; the number of IgE responders to r*Ov-*FAR-1 and to r*Ov-*FAR-2 antigens in the PI was 58.3% and 33.3%, respectively (Fig 6C and 6D), while the number of IgE responder in the INF was 76.7% and 46.7%, respectively (Fig 6E). In comparison, only the number of the IgG3 responders to r*Ov-*FAR-2 antigen in the infected (INF) or the exposed but uninfected (EN) children from the *W*. *bancrofti* endemic region was higher; 90.4% and 72.7%, respectively. The number of IgG1 responders to r*Bm-*FAR-1 and to r*Bm-*FAR-2 antigens was only 50%-66.6%, while the number of IgG3 responders to r*Bm-*FAR-1 antigens was 40.9–66.6%. No IgE responses were detected against any of the r*Bm-*FAR-1 and to r*Bm-*FAR-2 in both populations.

### Immunization with r*Bm-*FAR-1 induced partial protective immunity against *B*. *malayi* infection in gerbils

**Fig 7:**
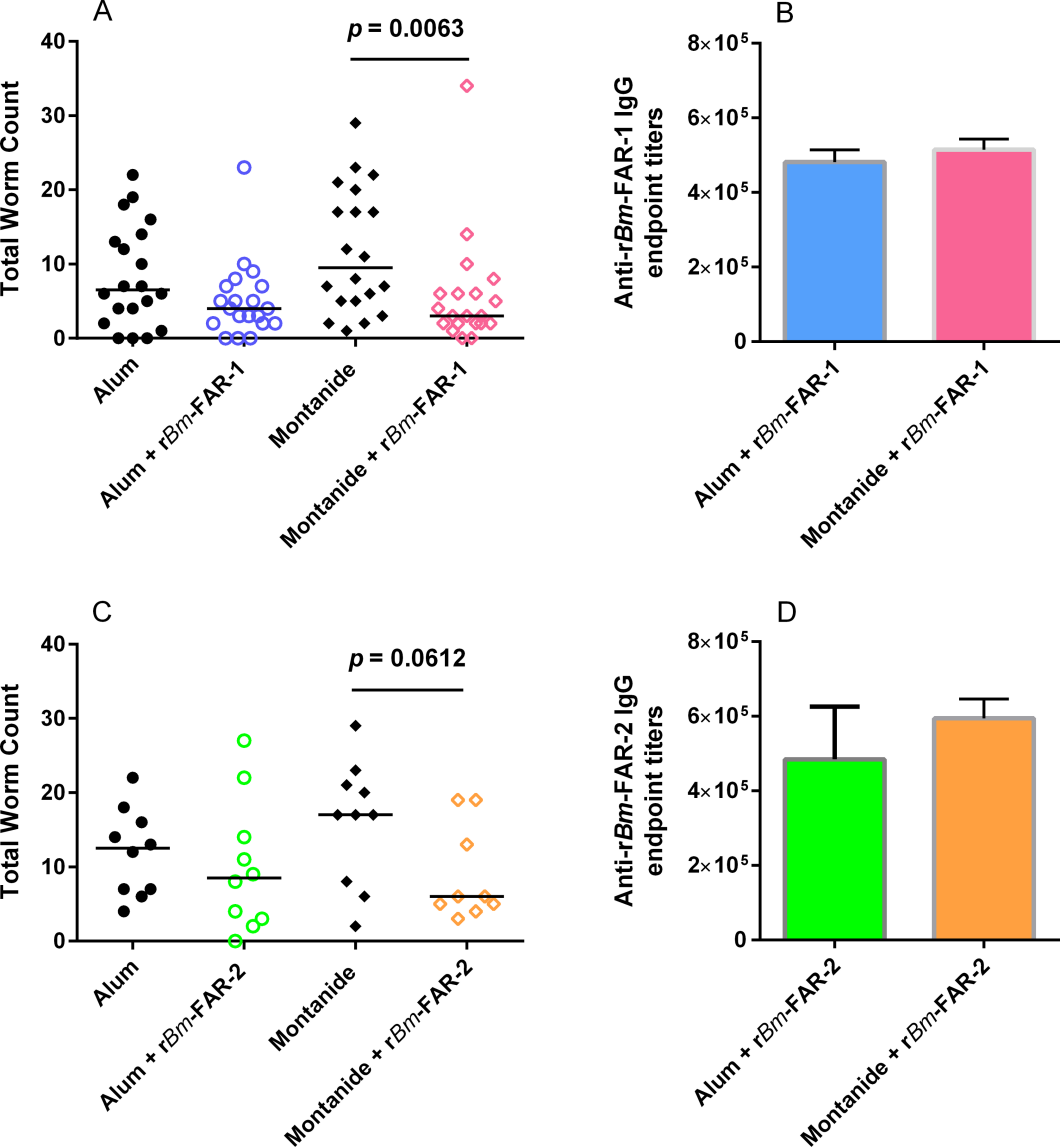
Protective immunity induced by immunization of gerbils with r*Bm-*FAR-1 and r*Bm-*FAR-2 formulated with Montanide-720 or alum. (**A)** Total worm count from gerbils (n = 20 per group, which are presented as a combination of two separate experiments) immunized with r*Bm-*FAR-1 formulated with alum or Montanide-720 or adjuvant controls 42 days after being challenged with 100 *B*. *malayi* L3. (**B)** Titers of anti-*Bm-*FAR-1 IgG in sera of gerbils immunized r*Bm-*FAR-1 formulated with alum or Montanide-720. (**C)** Total worm count from gerbils (n = 10 per group performed only once) immunized with r*Bm-*FAR-2 formulated with alum or Montanide-720 or adjuvant controls. (**D)** Titers of anti-*Bm-*FAR-2 IgG in sera taken from gerbils immunized with r*Bm-*FAR-2 formulated with alum or Montanide-720. The median in A and C is marked by a line. Geometric mean and 95% CI are marked in B and D.

After immunization with 25 μg of r*Bm-*FAR-1 formulated with alum or Montanide-720 for three times, and following challenge with 100 infective *B*. *malayi* L3, a significant reduction in worm numbers (68.4%, *p* = 0.0063) was observed only in the group of gerbils immunized with r*Bm-*FAR-1 formulated with Montanide-720 (Fig 7A). After immunization with 25 μg of r*Bm-*FAR-1 formulated with alum or Montanide-720 for three times, and following challenge with 100 infective *B*. *malayi* L3 in two separated experiments, a combined total worm reduction of 68.4% was observed with statistical significance (*p* = 0.0063) in the group of gerbils immunized with r*Bm-*FAR-1 formulated with Montanide-720 compared to adjuvant control group (Fig 7A). The worm reductions for individual r*Bm*-FAR-1/Montanide-720 immunization were 68.0% (*p* = 0.0139) and 44.4% (*p* = 0.0541), respectively, compared to adjuvant control. Though a trend of reduction (38.5%) was observed with the alum formulation, it was not statistically significantly different (*p* = 0.1458) from its corresponding alum control (Fig 7A). The efficacy outcomes have been assembled from two experiments done in separate times. The differences in protection between the two vaccine formulation cannot be simply accounted for by the overall anti-IgG r*Bm-*FAR-1 antibody titers; the titers in both vaccinated groups after three immunizations and before challenge were similar (geometric mean of 4.81 x 10^5^ and 5.15 x 10^5^, in alum or Montanide-720 formulated r*Bm-*FAR-1 vaccines, respectively) (Fig 7B).

Under the same immunization regimen, gerbils immunized with r*Bm-*FAR-2 formulated with alum or Montanide-720 only produced 16% and 42% of adult worm reduction, respectively, neither was statistically significant (*p* = 0.402 and *p* = 0.0612, respectively) compared with the respective adjuvant groups (Fig 6C, Table 1). In both immunized groups high antibody titers against r*Bm-*FAR-2 were nevertheless detected (geometric mean of 4.84 x 10^5^ and 5.94 x 10^5^, respectively) (Fig 6D).

**Table 1:** *B*. *malayi* adult worm recovery from gerbils immunized with adjuvant-formulated r*Bm*-FAR-1 or r*Bm*-FAR-2.

Vaccine	Experiment Number	Number of animals	Adjuvant	Mean worm ± SD		% Worm reduction	*p* value
				Adjuvant	Vaccine		
r*Bm*-FAR-1	1	10	Alum	4.7 ± 5.9	2.8 ± 2.8	40.4	0.6924
		10	Montanide	7.5 ± 6.2	2.4 ± 1.6	68.0*	0.0139*
	2	10	Alum	11.9 ± 5.8	7.4 ± 5.9	37.8	0.0546
		10	Montanide	16.0 ± 8.3	8.8 ± 9.6	44.4	0.0541
r*Bm*-FAR-2	3	10	Alum	11.9 ± 5.8	10.0 ± 8.8	16.0	0.402
		10	Montanide	16 ± 8.3	8.9 ± 6.4	42.0	0.0612

* Statistically significant, Mann–Whitney U Test, *p < 0*.*05*

## Discussion

The unusual ligand binding properties and nematode-specific structures set FAR proteins apart from mammalian lipid-bind proteins [5, 7]. The objectives of this study were to assess the structural and ligand-binding characters of two FAR proteins from the filarial parasite *B*. *malayi*, and evaluate their potential as vaccine candidates in gerbils. This is the first FAR-2 protein from a parasitic nematode that is being characterized.

The binding properties of r*Bm-*FAR-1 in this study were consistent with previously described results [2] in that r*Bm-*FAR-1 was able to bind retinol and DAUDA. While r*Bm-*FAR-2 also bound retinol, in unexpected contrast to r*Bm-*FAR-1, it did not produce the dramatic change in DAUDA’s emission observed with r*Bm*-FAR-1. Both proteins, however, bound cPnA, an unmodified intrinsically fluorescent fatty acid (Fig 5). The difference between the ligand binding propensities of the two proteins is surprising given previous experience with orthologous proteins in another species [7]. While there is no direct evidence of where in the two proteins retinol and fatty acids localize, the predicted tertiary structures of both proteins reveal differences that may explain the differential binding properties. *Bm-*FAR-1 and *Bm-*FAR-2 have similar putative retinol-binding pockets (P1), but *Bm-*FAR-2 has a smaller and less accessible predicted cavity for fatty acid binding (P2) than *Bm-*FAR-1. These possible structural differences are therefore consistent with the empirical finding that both proteins bind retinol similarly, but that r*Bm-*FAR-2 is more selective in its binding of fatty acids. In addition to the difference in structure and binding activities, *Bm-*FAR-1 is more abundant in all the stages of *B*. *malayi* than *Bm-*FAR-2, especially in the adult male adult worms and L4 larval stages, suggesting that *Bm-*FAR-1 may play quite different role(s) in the maintenance of life cycle than *Bm-*FAR-2. In *A*. *lumbricoides*, a FAR protein named ABA-1 is a major allergen was found to bind retinol and a range of fatty acids, including the pharmacologically active lipids lysophosphatidic acid, lysoplatelet activating factor, and leukotrienes [43]. To investigate whether these proteins are also immunoregulatory, it will be of interest to use the fluorescence-based assay used here to screen both FAR proteins (and their orthologues in *O*. *volvulus* and other species) for binding to immunologically- and inflammatory-related lipids such as leukotrienes and prostaglandins, or their precursors such as arachidonic acid.

We found that both *B*. *malayi* and *O*. *volvulus* FAR proteins are the targets of strong cytophilic IgG1 and IgG3 antibody responses in infected humans, as well as in those individuals classified as being protected (the EN and PI individuals). There is a difference between an antigen being the target of immune responses in infected and naturally immune individuals and their immunoprotective activity. We therefore proceeded to test the immunoprotective activity of the *Bm-*FAR proteins in gerbils. The results of this indeed suggested that their vaccine potential merits more extensive exploration, but also that *Bm-*FAR-1 and -2 may perform different functions in parasite survival. Gerbils immunized with r*Bm-*FAR1 elicited statistically significant protection against challenge of *B*. *malayi* L3, although this was dependent on the adjuvant used. Gerbils immunized with r*Bm-*FAR-2, on the other hand, were less protected, regardless of which adjuvant was used, despite the IgG antibody titers induced against either protein or adjuvant formulation being similar. The value of FAR proteins in protective immunity has also been observed in immunization trials with a FAR homologue from *O*. *volvulus* (*Ov*-FAR-1) against heterologous challenge of rodent filaria *A*. *viteae* in Mongolian gerbils [44], and from the human and animal hookworms [45]. The mechanism underlying the protection and the difference in the vaccine efficacy of the two proteins remains to be determined. The difference in their ligand binding properties and the expression abundance between *Bm-*FAR-1 and *Bm-*FAR-2 suggests both FARs may function differently in the maintenance of the parasite’s life cycle and interaction with host. The *Bm-*FAR-1 protein is more abundant after molting of L3, by twofold, which may indicate that it is more vulnerable to immunological attack than *Bm-*FAR-2. It cannot be excluded that the immuno-protective difference between r*Bm*-FAR-1 and r*Bm*-FAR-2 may partially results from the different expression system, which will need to be further confirmed in future experiments. In both *Bm-*FAR-1 and *Bm-*FAR-2 immunization, Montanide-720 adjuvant induced better protection than did alum. Montanide-720 is a water-in-oil emulsion preparation that induces strong and broad Th1- and Th2-associated humoral and cellular immune responses [46, 47], while alum is known to stimulate a Th2-type humoral and cellular response [48]. The adjuvants used here may therefore elicit different immunoglobulin isotype dominances (e.g. different IgG isotype balances, or IgE) and T cell response biases that may contribute to the difference of protection. Due to the lack of reagents for detecting IgG subclasses and cytokines in gerbils, the nature and biases of immune response induced with different adjuvants remains to be examined. Protection against filarial infection in humans appears to be associated with a combination of IgG1, IgG3 and IgE antibody responses as well as Th1-and Th2 immune responses to larval proteins [49–51].

In conclusion, we demonstrated that *Bm-*FAR-1 and *Bm-*FAR-2 are two FAR proteins with a similar but slightly different structure, exhibit different expression abundance and lipid-binding characteristics, and confer different levels of protection against *B*. *malayi* infection. We found a similar dichotomy in ligand binding between the orthologous proteins in *O volvulus*, which may unexpectedly apply widely amongst filarial nematode parasites. *B*. *malayi* FARs, especially *Bm-*FAR-1 may play important roles in the survival of the filarial parasite in the host, therefore are promising vaccine candidates for a prophylactic vaccine against lymphatic filariasis.

## Supporting information

S1 Fig Expression of r*Bm-*FAR-2 with His-tag in *E*. *coli* BL21 and purified with IMAC. Lane 1, SDS-PAGE of purified r*Bm-*FAR-2 (2 μg). Lane 2, Western blot of 100 ng of r*Bm-*FAR-2 probed with anti-His antibody (1:3,000).
(TIF)

S2 Fig Similarities in the hydrophobic ligand-binding properties of FAR-1 and FAR-2 proteins of *B*. *malayi* with orthologous FARs of *O*. *volvulus*. Fluorescence emission spectra were recorded, and spectrofluorimetry conditions set, as described in Materials and Methods. The ligands were all-trans retinol (**A**), dansylamino undecanoic acid (DAUDA), a fatty acid conjugated to the environmentally sensitive dansyl fluorophore (**B**), and, cis-parinaric acid (cPnA), a natural fatty acid that is intrinsically fluorescent, the emission of which is, like dansyl, environment-sensitive (**C**). All of the proteins were estimated to be at concentrations of ~4 mg ml^-1^. These results show that, firstly, the proteins from both parasites bind retinol, though FAR-2s elicit less of a fluorescence enhancement of retinol than FAR-1s in these experimental conditions (**A**). Second, that FAR-1s bind DAUDA, yielding similarly substantial increases and blue shift in its peak fluorescence emission, but FAR-2s bind elicit only minor changes in DAUDA fluorescence (**B**). As said in the main text, this could be because either FAR-2s bind DAUDA poorly, or that they bind the fatty acid moiety of DAUDA but leave the attached dansyl fluorophore exposed to a polar environment. Third, both FAR-1s and FAR-2s bind a natural, intrinsically fluorescent, fatty acid acid (cPnA), indicating that all of these proteins bind fatty acids (**C**). As with retinol, the FAR-2s elicited weaker changes in the emission of cPnA than the FAR-1s. Note that *Bm-*FAR-2 is larger than *Bm-* FAR-1, such that at equivalent w/v concentrations their molarities will differ. Also, we cannot assume that the proportion of properly folded and active protein in each protein sample is equivalent, or that there is no interference from resident ligand(s) derived from the bacteria in which the proteins were produced.
(TIF)
