# Supplementary figures and images for: Ligand binding properties of two Brugia malayi fatty acid and retinol (FAR) binding proteins and their vaccine efficacies against challenge infection in gerbils
Source: PLoS Negl Trop Dis. 2018 Oct 8;12(10):e0006772. doi: 10.1371/journal.pntd.0006772 (PMC6193737; doi:10.1371/journal.pntd.0006772)

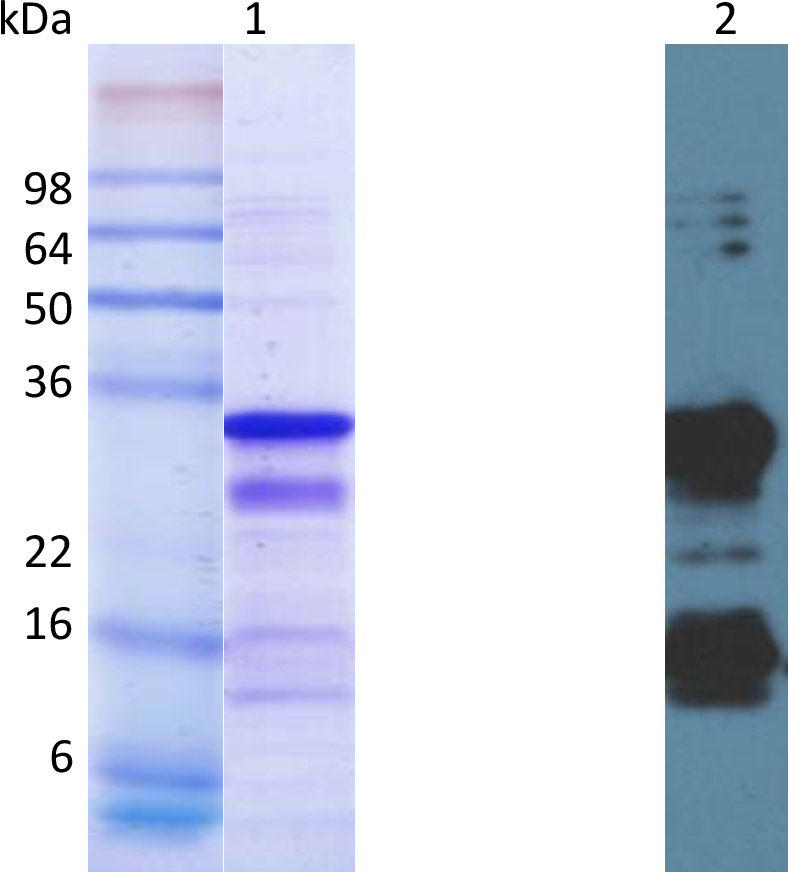

Supplement: S1 Fig — Lane 1, SDS-PAGE of purified rBm-FAR-2 (2 μg). Lane 2, Western blot of 100 ng of rBm-FAR-2 probed with anti-His antibody (1:3,000). (TIF) [file pntd.0006772.s001.tif]

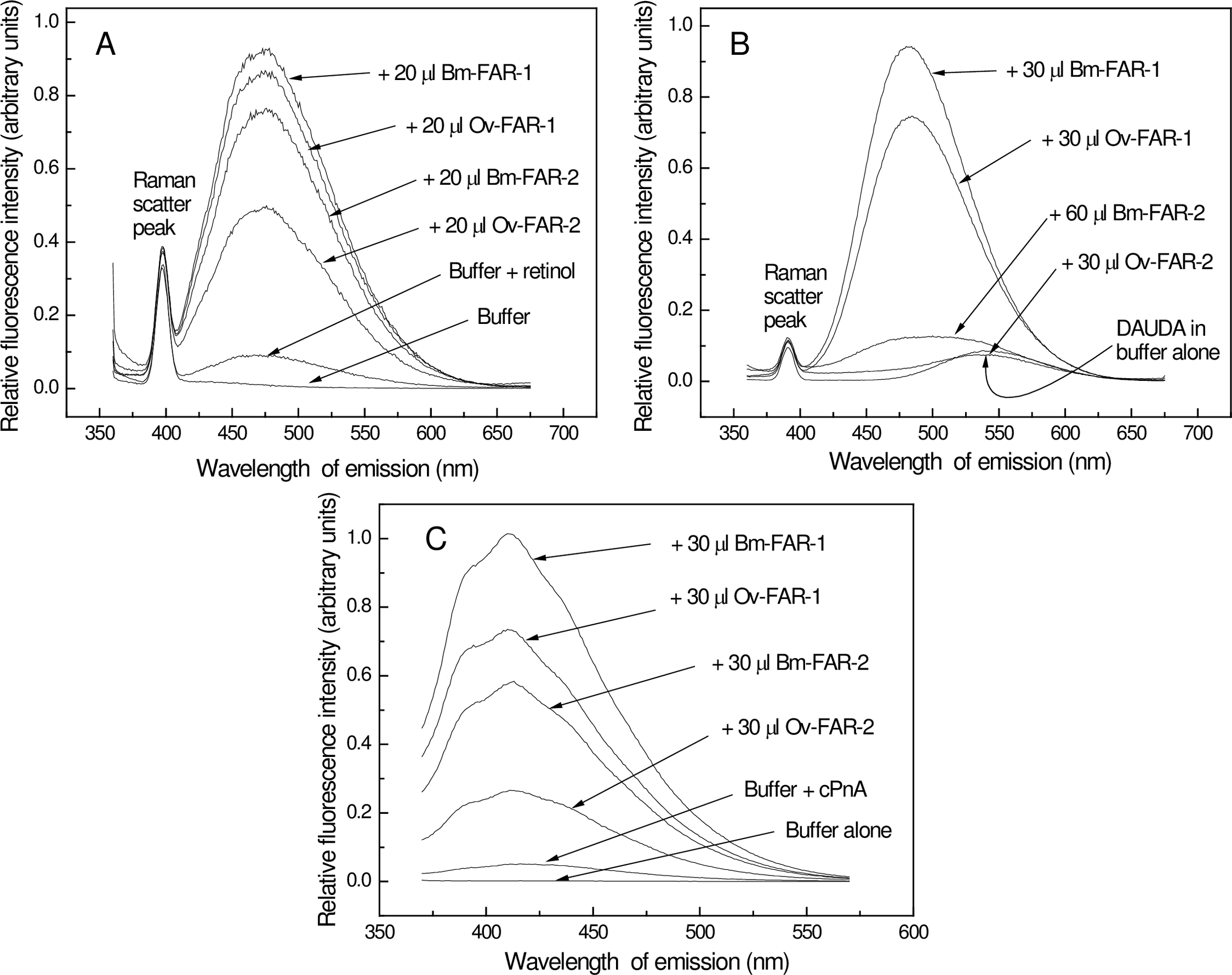

Supplement: S2 Fig — Fluorescence emission spectra were recorded, and spectrofluorimetry conditions set, as described in Materials and Methods. The ligands were all-trans retinol (A), dansylamino undecanoic acid (DAUDA), a fatty acid conjugated to the environmentally sensitive dansyl fluorophore (B), and, cis-parinaric acid (cPnA), a natural fatty acid that is intrinsically fluorescent, the emission of which is, like dansyl, environment-sensitive (C). All of the proteins were estimated to be at concentrations of ~4 mg ml-1. These results show that, firstly, the proteins from both parasites bind retinol, though FAR-2s elicit less of a fluorescence enhancement of retinol than FAR-1s in these experimental conditions (A). Second, that FAR-1s bind DAUDA, yielding similarly substantial increases and blue shift in its peak fluorescence emission, but FAR-2s bind elicit only minor changes in DAUDA fluorescence (B). As said in the main text, this could be because either FAR-2s bind DAUDA poorly, or that they bind the fatty acid moiety of DAUDA but leave the attached dansyl fluorophore exposed to a polar environment. Third, both FAR-1s and FAR-2s bind a natural, intrinsically fluorescent, fatty acid acid (cPnA), indicating that all of these proteins bind fatty acids (C). As with retinol, the FAR-2s elicited weaker changes in the emission of cPnA than the FAR-1s. Note that Bm-FAR-2 is larger than Bm- FAR-1, such that at equivalent w/v concentrations their molarities will differ. Also, we cannot assume that the proportion of properly folded and active protein in each protein sample is equivalent, or that there is no interference from resident ligand(s) derived from the bacteria in which the proteins were produced. (TIF) [file pntd.0006772.s002.tif]
